# Betulinic Acid Ameliorates the T-2 Toxin-Triggered Intestinal Impairment in Mice by Inhibiting Inflammation and Mucosal Barrier Dysfunction through the NF-κB Signaling Pathway

**DOI:** 10.3390/toxins12120794

**Published:** 2020-12-13

**Authors:** Chenxi Luo, Chenglong Huang, Lijuan Zhu, Li Kong, Zhihang Yuan, Lixin Wen, Rongfang Li, Jing Wu, Jine Yi

**Affiliations:** 1 Hunan Engineering Research Center of Livestock and Poultry Health Care, Colleges of Veterinary Medicine, Hunan Agricultural University, Changsha 410128, China; luochenxione@stu.hunau.edu.cn (C.L.); huangchenglongedu@hotmail.com (C.H.); zhulijuan@stu.hunau.edu.cn (L.Z.); kongli@stu.hunau.edu.cn (L.K.); zhyuan2016@hunau.edu.cn (Z.Y.); wenlixinedu@hotmail.com (L.W.); lirongfang@hunau.edu.cn (R.L.); 2 Hunan Co-innovation Center of Animal Production Safety, Changsha 410128, China

**Keywords:** T-2 toxin, betulinic acid, intestine, oxidative damage, NF-κB signaling pathway

## Abstract

T-2 toxin, a trichothecene mycotoxin produced by *Fusarium*, is widely distributed in crops and animal feed and frequently induces intestinal damage. Betulinic acid (BA), a plant-derived pentacyclic lupane-type triterpene, possesses potential immunomodulatory, antioxidant and anti-inflammatory biological properties. The current study aimed to explore the protective effect and molecular mechanisms of BA on intestinal mucosal impairment provoked by acute exposure to T-2 toxin. Mice were intragastrically administered BA (0.25, 0.5, or 1 mg/kg) daily for 2 weeks and then injected intraperitoneally with T-2 toxin (4 mg/kg) once to induce an intestinal impairment. BA pretreatment inhibited the loss of antioxidant capacity in the intestine of T-2 toxin-treated mice by elevating the levels of CAT, GSH-PX and GSH and reducing the accumulation of MDA. In addition, BA pretreatment alleviated the T-2 toxin-triggered intestinal immune barrier dysregulation by increasing the SIgA level in the intestine at dosages of 0.5 and 1 mg/kg, increasing IgG and IgM levels in serum at dosages of 0.5 and 1 mg/kg and restoring the intestinal C3 and C4 levels at a dosage of 1 mg/kg. BA administration at a dosage of 1 mg/kg also improved the intestinal chemical barrier by decreasing the serum level of DAO. Moreover, BA pretreatment improved the intestinal physical barrier via boosting the expression of ZO-1 and Occludin mRNAs and restoring the morphology of intestinal villi that was altered by T-2 toxin. Furthermore, treatment with 1 mg/kg BA downregulated the expression of p-NF-κB and p-IκB-α proteins in the intestine, while all doses of BA suppressed the pro-inflammatory cytokines expression of IL-1β, IL-6 and TNF-α mRNAs and increased the anti-inflammatory cytokine expression of IL-10 mRNA in the intestine of T-2 toxin-exposed mice. BA was proposed to exert a protective effect on intestinal mucosal disruption in T-2 toxin-stimulated mice by enhancing the intestinal antioxidant capacity, inhibiting the secretion of inflammatory cytokines and repairing intestinal mucosal barrier functions, which may be associated with BA-mediated inhibition of the NF-κB signaling pathway activation.

## 1. Introduction

T-2 toxin is a trichothecene mycotoxin produced in nature by *Fusarium* fungi which gives rise to animal feed and food contamination and instigates mass poisoning around the world, resulting in serious economic losses to agriculture and animal husbandry in countries worldwide [1,2,3]. The ingestion of T-2 toxin causes vomiting, feed refusal, growth retardation, immune system and digestive system disorders, potential harm to human and animal health and issues with animal-derived food safety [4,5]. By accumulating in organisms and being transported through food chains, and finally delivering to animals and humans, the cytotoxicity of T-2 toxin attracted great attention. As the intestinal barrier is the first defense against external stimuli, the intestinal mucosa is composed of physical, chemical, immunological and biological barriers [6]. T-2 toxin mainly causes intestinal mucosal barrier dysfunction by inducing inflammation and oxidative stress [7]. Therefore, the identification of a natural, active substance that alleviates the intestinal damage caused by T-2 toxin is very important to improve food safety and animal product quality.

Betulinic acid (BA), a pentacyclic triterpenoid, is primarily present in food, fruits and medicinal herbs, particularly in white birch bark [8]. BA possesses an array of pharmacological properties, such as antioxidative stress, anti-inflammatory, antitumor and antiviral activities [9,10]. Even a high dose of BA at 500 mg/kg is generally recognized as safe in athymic mice [11,12]. The antioxidation and anti-inflammation of BA have been extensively explored in vivo and in vitro [13,14]. BA reduces the contents of interleukin (IL)-β, IL-6, tumor necrosis factor (TNF)-α and malondialdehyde (MDA) in the kidney and synovial cells of diabetic rats with rheumatoid arthritis, enhances the levels of the antioxidant enzymes superoxide dismutase (SOD) and catalase (CAT) and inhibits the phosphorylation of nuclear factor-kappa B (NF-κB) p65 and inhibitor of NF-κB (IκB), thereby exerting anti-inflammation and antioxidant properties through activation of the nuclear factor erythroid-2-related factor 2 (Nrf2) pathway and inhibition of the NF-κB signaling pathway [15,16].

In a previous study, it has been showed that BA protected against cyclophosphamide (CYP)-caused intestinal mucosal injury [17]. However, relatively few studies have explored the protective effect of BA on intestinal mucosal barrier disruption challenged by T-2 toxin, and its possible mechanism remains poorly understood. In this study, we induced an intestinal mucosal oxidative lesion by a single injection of T-2 toxin to inquire about the preventive effect of BA against intestinal impairment in T-2 toxin-exposed mice. Thus, we were able to detect the effects of pretreatment with BA on the degree of oxidative stress, mucosal barrier integrity, inflammatory responses and the expression of related proteins in the NF-κB signaling pathway in the intestine of T-2 toxin-exposed mice using Western blotting, quantitative real-time PCR (qPCR), hematoxylin-eosin (H&E) staining and transmission electron microscopy (TEM). This could provide a regulatory target for relieving intestinal impairment caused by T-2 toxin and exploring the feasibility of BA in intestinal regulation and nutritional intervention.

## 2. Results

### 2.1. BA Reduced Intestinal Oxidative Stress Triggered by T-2 Toxin in Mice

**Figure 1:**
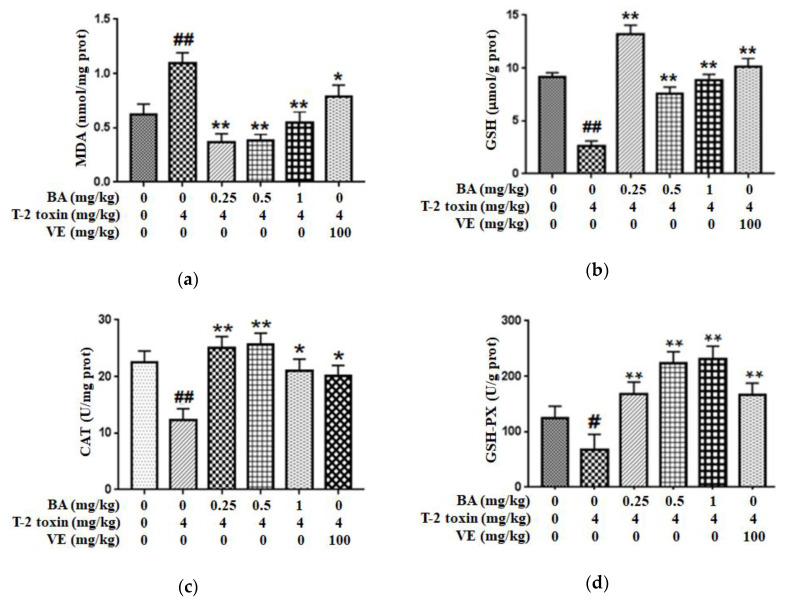
Effect of BA on the level of lipid peroxidation marker MDA (**a**), and the antioxidant capacity of GSH (**b**), CAT (**c**) and GSH-PX (**d**) in the intestine of T-2 toxin-treated mice. Values are presented as the mean ± standard deviation (SD) in each treatment. “#” *p* < 0.05 and “##” *p* < 0.01 compared to the control group; “*” *p* < 0.05 and “**” *p* < 0.01 compared to the T-2 group.

In order to assess the possible protective role of BA against intestinal impairment associated with T-2 toxin administration, the oxidative damage-related indicators were detected. First, we explored whether BA pretreatment attenuated T-2 toxin-triggered oxidative stress by measuring the levels of the lipid peroxidation marker (MDA) and an antioxidant (reduced glutathione (GSH)), and the antioxidant activities of glutathione peroxidase (GSH-PX) and CAT in the intestine (Figure 1). From the results, compared with the control group, T-2 toxin significantly increased the MDA content and decreased the CAT, GSH and GSH-PX levels in the intestine. Meanwhile, vitamin E (VE), as a positive control, and BA pretreatment alleviated the decreases in the CAT, GSH-PX and GSH levels in the intestine exposed to T-2 toxin and decreased the generation of MDA, thereby improving the antioxidant capacity of the intestine.

### 2.2. BA Prevented the Intestinal Morphological Changes Caused by T-2 Toxin in Mice

**Figure 2:**
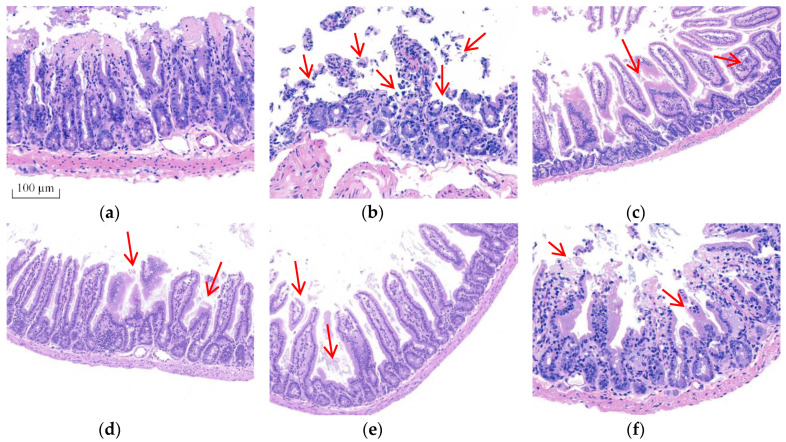
Effect of BA on the morphological structure of the intestine in T-2 toxin-treated mice analyzed using H&E staining. Notes: (**a**), control group; (**b**), T-2 toxin group; (**c**), 0.25 mg/kg of BA group; (**d**), 0.5 mg/kg of BA group; (**e**), 1 mg/kg of BA group; (**f**), VE group. Red arrows show the breaking, loss and irregular arrangement of intestinal villi. Scale bar: 100 μm.

**Figure 3:**
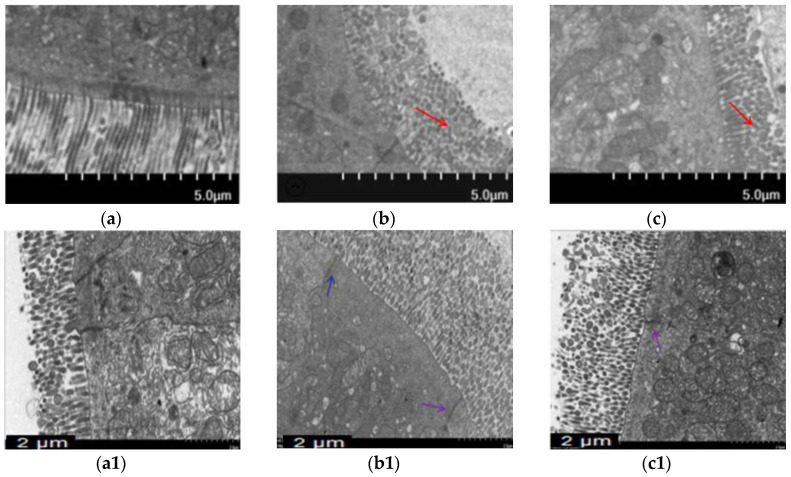
Effect of BA on intestinal microvilli and tight junction structures in mice processed with T-2 toxin analyzed using TEM. Notes: (**a**), intestinal microvilli in the control group; (**b**), intestinal microvilli in T-2 toxin group; (**c**), intestinal microvilli in 0.5 mg/kg BA group. The red arrow indicates broken and shorter intestinal microvilli. Scale bar: 5.0 μm. (**a1**), intestinal tight junction structure in the control group; (**b1**), intestinal tight junction structure in T-2 toxin group; (**c1**), intestinal tight junction structure in the 0.5 mg/kg BA group. The blue arrow indicates the intestinal tight junction structure. Scale bar: 2 μm.

Morphological changes were analyzed using H&E staining (Figure 2) and ultrastructural changes in intestine were investigated using TEM (Figure 3) to prove BA’s protective effect on the intestine. The intestinal villi were arranged clearly and regularly in the control group. The microvilli were arranged compactly, and the size of cells was approximately the same, forming an integral tight junction. Meanwhile, after intraperitoneal injection of T-2 toxin, a thickening and shortening of intestinal microvilli and villi occurred, as well as a disruption of the tight junction structure. Furthermore, the villi were broken or dissolved to varying degrees, with a loose arrangement between the intestinal villus. After the BA pretreatment, villus heights were increased, and the morphological structures of villi and tight junctions were significantly improved.

### 2.3. BA Increased the Tight Junction Proteins (TJs) mRNA Expressions in Intestine of T-2 Toxin-Intoxicated Mice

**Figure 4:**
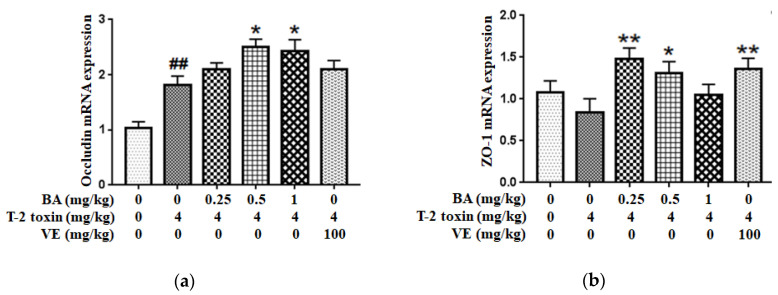
Effect of BA on Occludin (**a**) and ZO-1 (**b**) mRNAs expressions in the intestine of T-2 toxin-treated mice. Values are presented as the mean ± SD in each treatment. “##” *p* < 0.01 compared to the control group; “*” *p* < 0.05 and “**” *p* < 0.01 compared to the T-2 group.

Next, the mRNA expressions of intestinal TJs (Figure 4) were measured to assess BA’s protective effect on the intestinal physical barrier in T-2 toxin-intoxicated mice. As the results show, T-2 toxin slightly lowered the ZO-1 mRNA expression and elevated the Occludin mRNA expression. However, BA pretreatment significantly accelerated the mRNA expressions of intestinal TJs.

### 2.4. BA Alleviated Intestinal Immune Barrier Impairment in T-2 Toxin-Triggered Mice

**Figure 5:**
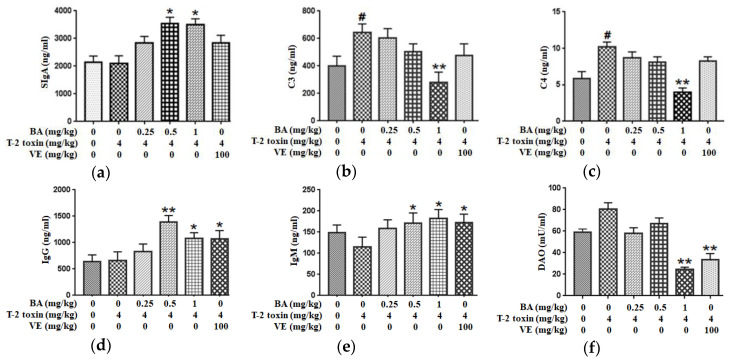
Effects of BA on the secretions of SIgA (**a**), C3 (**b**) and C4 (**c**) in the jejunum and the levels of IgG (**d**), IgM (**e**) and diamine oxidase (DAO) (**f**) in the serum processed with T-2 toxin in mice. Values are presented as the mean ± SD in each treatment. “#” *p* < 0.05 compared to the control group; “*” *p* < 0.05 and “**” *p* < 0.01 compared to the T-2 group.

We measured the levels of secretory immunoglobulin A (SIgA), complement 3 (C3) and C4 in the jejunum and the serum levels of immunoglobulin M (IgM) and IgG to evaluate the immune barrier function of the intestinal mucosa. Compared to the control group, T-2 toxin promoted the secretion of C3 and C4 in the intestine, with no effect on SIgA secretion in the intestine. Pretreatment with 1 mg/kg BA, however, markedly reduced the production of C3 and C4. Additionally, administration of BA significantly increased the SIgA level (Figure 5a–c). At the same time, T-2 toxin declined the serum IgM concentration (*p* > 0.05), with no effect on the serum IgG level. However, medium- and high-dose BA pretreatments obviously increased serum levels of IgG and IgM. Similarly, VE pretreatment markedly upregulated the IgG and IgM levels (Figure 5d,e). Thus, BA or VE improves the intestinal immune barrier function of T-2 toxin-treated mice. 

### 2.5. BA Decreased the Serum DAO Activity in Mice Exposed to T-2 Toxin

DAO is a highly active intracellular enzyme in the upper villi of the small intestinal mucosa, and the DAO level in serum is closely related to the intestinal chemical barrier. T-2 toxin enhanced DAO activity in serum (*p* > 0.05). After the high dosage of BA or VE pretreatment, DAO activity was decreased significantly (Figure 5f). Based on the findings, BA or VE alleviate the intestinal chemical barrier dysfunction in T-2 toxin-exposed mice.

### 2.6. BA Regulated T-2 Toxin-Triggered Inflammatory Cytokine Secretion in the Intestine

**Figure 6:**
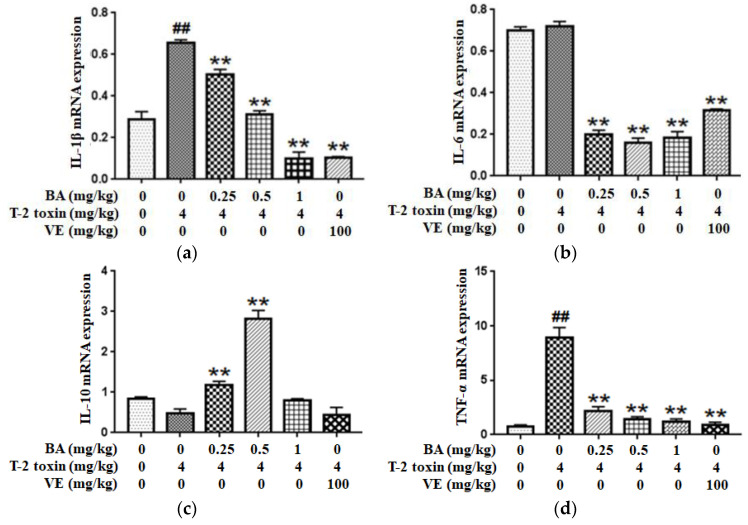
Effect of BA on the IL-1β (**a**), IL-6 (**b**), IL-10 (**c**) and TNF-α (**d**) mRNAs expressions in the intestine of T-2 toxin-exposed mice. Values are presented as the mean ± SD in each treatment. “##” *p* < 0.01 compared to the control group; “**” *p* < 0.01 compared to the T-2 group.

Since inflammation plays a key role in T-2 toxin-evoked intestinal mucosal barrier dysfunction in mice, the expressions of inflammatory cytokines such as IL-1β, IL-6, IL-10 and TNF-α were examined using qPCR to explore whether the BA pretreatment ameliorated T-2 toxin-induced intestinal inflammation. As shown in Figure 6a,d, compared with the control group, the pro-inflammatory cytokines IL-1β and TNF-α have higher expression levels in the T-2 toxin group, while the level of IL-6 had no significant effect. However, the levels were markedly decreased after the pretreatment with BA. Conversely, as shown in Figure 6c, mice pretreated with BA presented significantly higher IL-10 levels in the intestine than mice in the T-2 toxin group. Thus, BA exhibits an excellent ability to attenuate the T-2 toxin-caused increase in inflammation.

### 2.7. BA Attenuated T-2 Toxin-Caused NF-κB Activation in the Intestine

**Figure 7:**
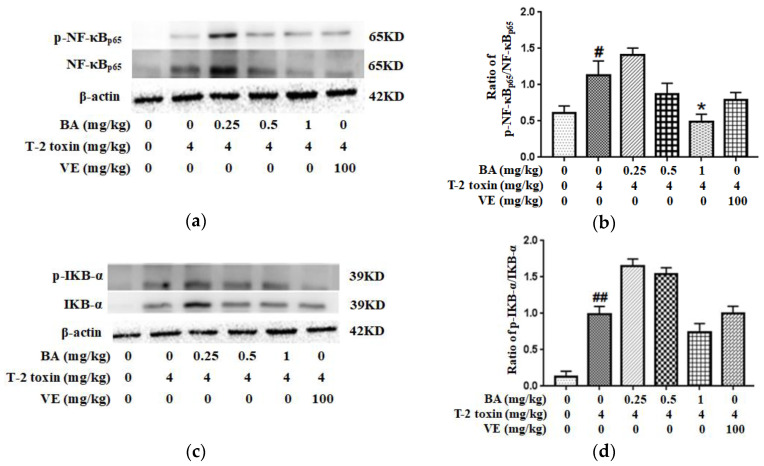
Effect of BA on the intestinal proteins expression involved in the NF-κB signaling pathway in T-2 toxin-intoxicated mice. Western blot was used to analyze the effect of BA on NF-κB (**a**) and IKB-α (**c**) levels. The data are presented as the ratios of p-NF-κB/NF-κB (**b**) and p-IKB-α/IKB-α (**d**). Values are presented as the mean ± SD in each treatment. “#” *p* < 0.05 and “##” *p* < 0.01 compared to the control group; “*” *p* < 0.05 compared to the T-2 group.

Western blotting was used to detect the levels of related proteins in the NF-κB pathway to further investigate the potential mechanism by which BA attenuated T-2 toxin-caused oxidative damage and inflammation in the intestine. It could be seen from the results that T-2 toxin increased the expression of p-NF-κB/NF-κB and p-IKB-α/IKB-α proteins in the intestine (Figure 7). Meanwhile, the high-dose BA decreased the ratio of p-NF-κB/NF-κB, while BA pretreatment had a slightly lower ratio of p-IKB-α/IKB-α (*p* > 0.05). Taken together, NF-κB is indispensable for the effects of BA on inhibiting intestinal mucosal barrier damage triggered by T-2 toxin.

## 3. Discussion

Trichothecene mycotoxins, including T-2 toxin, widely exist in moldy feed materials and human food. Animals ingesting feed contaminated with T-2 toxin or humans taking food contaminated with T-2 toxin present a serious health hazard [18]. The intestine is a remarkable digestive and immune organ responsible for the digestion of food, absorption of energy, providing nutritional substances and generating immune responses [19]. T-2 toxin causes symptoms of poisoning mainly from the absorption of the intestinal tract. Therefore, the intestine is more vulnerable to toxic damage that causes intestinal mucosal barrier dysfunction [20]. As the first barrier providing defense against stimuli from the external environment, the intact intestinal mucosa plays an important role in maintaining intestinal homeostasis and normal intestinal physiological function [21]. The intestinal mucosa is protected by a mechanical barrier consisting of the TJs and adherens junction proteins of epithelial cells, a chemical barrier provided by antimicrobial peptides, gastric acid and lysozyme, an immunological barrier that includes cells and molecules of the innate and adaptive immune system and a microbiological barrier that prevents the migration of microbial contaminants [22]. BA, a pentacyclic triterpene, is widely found in birch bark and many plants and has been considered a natural antioxidant and anti-inflammatory agent [9]. BA protects against dextran sulfate sodium (DSS)-induced colitis and prophylactically protects against CYP-induced intestinal mucosal barrier damage by improving the antioxidant capacity and reducing lipid peroxidation [17,23], indicating that BA might be an effective new drug to alleviate intestinal damage. To date, researchers have not investigated the effect of BA on T-2 toxin-triggered impairment of the intestinal mucosal barrier. Hence, we investigated the preventive protection of BA on T-2 toxin-evoked oxidative stress, inflammation, mucosal barrier damage and the NF-κB signaling pathway in the intestine to further clarify the potential mechanisms by which BA ameliorates intestinal mucosal barrier dysfunction.

Oxidative stress participates in the initiation and development of cardiovascular and digestive diseases and is considered as the underlying mechanism of intestinal barrier dysfunction [24]. The underlying mechanism of T-2 toxin-triggered cell apoptosis and tissue damage was shown to be oxidative stress, which was partially regulated by the overproduction of ROS, inhibition of the activity of antioxidant enzymes and an increase in lipid peroxidation [25]. These findings are similar to the results of the present study showing that T-2 toxin remarkably declined the levels of CAT, GSH and GSH-PX and elevated the accumulation of MDA in the intestine. Meanwhile, the BA supplement improved antioxidase activities and decreased the MDA accumulation in the intestine of T-2 toxin-exposed mice (Figure 1). Similar studies reported that BA markedly reduced the MDA level and restored antioxidant enzyme activities in rat models of experimental membranous nephropathy or diabetic nephropathy [15,26]. Dietary BA may alleviate CYP-induced lipid peroxidation in the intestine [17]. Moreover, BA significantly prevents diarrhea, bleeding and colonic pathological changes in DSS-exposed mice via reducing the accumulation of MDA, myeloperoxidase and lipid hydroperoxide and increasing SOD and CAT activities to normalize the redox balance in the colon [23]. BA was suggested to exert a preventive protection on intestinal mucosal damage in part by improving the antioxidant capacity of the intestine.

The intestinal mucosal mechanical barrier is the structural foundation for maintaining the intestinal epithelium and its barrier function. TJs formed by proteins such as ZO-1, Claudin-1 and Occludin between intestinal epithelial cells play a pivotal part in keeping the epithelial cell structure, thus preserving the biological function of the intestinal barrier [27,28]. Abnormal expression of TJs increases the selective permeability of the intestinal epithelium, resulting in mechanical barrier dysfunction, which is one of the characteristics of intestinal disease [29,30]. The mRNA expression of TJs and the morphological alterations of intestinal villi were observed to evaluate whether BA ameliorated the T-2 toxin-evoked intestinal mechanical barrier disruption. In the present study, ZO-1 mRNA expression was lower, while Occludin mRNA expression was enhanced in the intestine of T-2 toxin-exposed mice (Figure 4). This finding conforms with the report by Liu et al. [31], who revealed that T-2 toxin downregulated the expression of ZO-1 and Occludin in the rabbit intestine. Based on accumulating evidence, the alterations in the mRNA expression of TJs caused by mycotoxins are not completely consistent with the levels of TJ proteins [32,33]. The expressions of Claudin-3, Claudin-4, Occludin and ZO-1 mRNAs were increased in deoxynivalenol-exposed Caco-2 cells, but the levels of these proteins were significantly reduced [34]. The discrepancy in the effect of T-2 toxin might be very closely related to the types of animals or cells analyzed and the sensitivity of TJs to various mycotoxins [35]. The expression of the Occludin mRNA was markedly increased in T-2-toxin-exposed mice, which was also possibly part of a counter mechanism to alleviate intestinal mechanical barrier dysfunction. The intestinal mucosal injury induced by T-2 toxin would eventually lead to a compensatory response involving stimulation of the expression of TJs, such as Occludin. However, administration of BA markedly upregulated the expression of Occludin and ZO-1 in the intestine. Furthermore, the morphological alterations of intestinal villi observed using H&E staining and TEM showed that BA significantly ameliorated T-2 toxin-induced shortening and thickening of intestinal villi, along with the destruction of the epithelial cell layer (Figure 2 and Figure 3). A similar study has showed that BA increased TJs expression, improved the intestinal morphological structure and inhibited the infiltration of inflammatory cells in CYP-challenged mice [17]. Thus, BA may attenuate the disruption of the intestinal mucosa mechanical barrier induced by T-2 toxin by improving the intestinal integrity and morphology.

DAO, an intracellular enzyme, is present in the cytoplasm of villi located in the intestinal stratum supravasculare, and its highest activity is detected in the intestinal mucosa in animals. When the intestinal mucosa is injured, the level of DAO is decreased due to its release from the intestinal mucosa and entry into the blood, ultimately leading to a higher level of DAO in blood plasma. Hence, the increase in serum DAO level is considered as a parameter of mucosal impairment and barrier permeability [36,37]. A similar finding was obtained in this experiment, which confirmed that T-2 toxin accelerated the release of DAO into the bloodstream and damaged the intestinal structure and function. After the high dosage of BA pretreatment, a significantly lower serum DAO level was detected than in the T-2 toxin group (Figure 5f). BA ameliorated the intestinal mucosal permeability, decreased the extent of intestinal mucosal lesion and substantially alleviated the disruption of intestinal barrier function.

The function of the intestinal immunological barrier primarily relies on immunoglobulins, complement proteins and cytokines [38]. Immunoglobulins such as IgM, IgG and secretory IgA are secreted by the intestinal mucosal lamina propria. They are the major humoral immune components and effector molecules in the intestinal mucosal surfaces. The complement system is an integral part of innate immunity in the organism and is associated with the initiation of adaptive immune reactions. In normal states, the complement system contributes to the natural defenses against pathogens, while improper activation of the complement system may result in tissue damage [39]. Mycotoxins disrupt the humoral and cellular immunity of the intestinal mucosa [40,41]. Exposure to T-2 toxin exerted an immunosuppressive effect by inhibiting the secretion of immunoglobulins and decreasing the levels of complement components in serum [42,43,44]. In our study, T-2 toxin had no obvious effect on the serum IgG and IgM levels and the intestinal SIgA level, suggesting that an acute exposure to T-2 toxin by intraperitoneal injection was not adequate to alter immunoglobulin levels. Meanwhile, T-2 toxin evoked the production of C3 and C4 in the intestine (Figure 5a–e). A potential explanation for this finding is that T-2 toxin causes inappropriate complement activation and the production of C3 and C4, which in turn induces the secretion of pro-inflammatory cytokines and intestinal mucosal damage. However, after the pretreatment with BA, the increase in intestinal C3 and C4 levels triggered by T-2 toxin was inhibited, the secretion of intestinal SIgA was promoted and the serum IgG and IgM levels were increased, indicating that BA may exert a protective effect on intestinal mucosal injury partly by inducing immunoglobulin secretion, maintaining the balance of complement components and modulating intestinal immunity.

In addition, inflammation plays a vital role in modulating the intestinal mucosal immune barrier function [45]. Inflammatory cytokines are regulated by the NF-κB signaling pathway. NF-κB, a key factor that induces the expression of inflammatory cytokines, commonly exists in the cytoplasm, where it binds IκB to the formation of an inactive complex. The nuclear translocation of p65 is the signal for NF-κB activation, and the phosphorylation and subsequent degradation of IκB lead to NF-κB activation which induces the transcription of pro-inflammatory cytokines, ultimately, in turn, activating NF-κB [46,47]. Excess production of inflammatory cytokines such as TNF-α and IL-1β may directly result in intestinal barrier injury [48,49]. Based on accumulating evidence, elevated generation of IL-1β, IL-6 and TNF-α increases local inflammation and promotes tissue damage induced by pathological stimuli, including intestinal barrier injury [50,51]. Mycotoxins promote the expression of pro-inflammatory cytokines in intestinal inflammatory diseases [52]. Therefore, the regulation of these inflammatory cytokines is an effective strategy to ameliorate intestinal injury. In the last several years, some studies have reported that phytogenic feed additives ameliorate inflammation by downregulating the NF-κB pathway, which is involved in the modulation of the intestinal immunological barrier in animals [51,53]. BA exerts a significant suppressive effect on inflammation, thereby inhibiting the secretion of cytokines induced by external stimuli [54]. BA markedly alleviates λ-carrageenan-induced paw edema by decreasing the serum levels of the IL-6 and IL-1β [23]. Moreover, BA pretreatment decreased the level of TNF-α and increased the production of anti-inflammatory cytokines such as IL-2 and IL-10 in serum, indicating the protective effects of BA on intestinal mucosal impairment in CYP-challenged mice [9]. Furthermore, BA inhibited the activation of NF-κB and subsequent gene expression induced by a lipopolysaccharide in RAW 264.7 macrophages [55]. The findings in the current study are similar to previous studies, as pretreatment with BA at a dose of 1 mg/kg suppressed the increase in NF-κB and IKB-a phosphorylation caused by T-2 toxin, and BA significantly decreased the generation of IL-1β, IL-6 and TNF-α and increased the secretion of IL-10 in the intestine of T-2 toxin-exposed mice (Figure 6 and Figure 7). Therefore, BA attenuated T-2 toxin-triggered intestinal barrier damage by controlling the inflammatory response in the intestine through the suppression of the NF-κB pathway. However, 0.25 and 0.5 mg/kg BA pretreatments had no obvious effect on the phosphorylation of the NF-κB and IKB-α proteins, and the potential explanations for the discrepancy are related to the tested doses, individual differences and the sensitivity to various mycotoxins, among other factors.

Based on these findings, BA exerts preventive and protective effects on T-2 toxin-triggered intestinal barrier dysfunction in mice by decreasing oxidative stress and repairing the physical, chemical and immune barrier functions of the intestinal mucosa, which are associated with inhibiting the activation of the NF-κB signaling pathway. The results may help to form the theoretical basis of the mechanism of BA and contribute a viable alternative to alleviate mycotoxin-induced intestinal injury to ensure and maintain intestinal health. In addition, the microbiota also plays a critical role in supporting the physiological functions and maintaining intestinal health, owing to its metabolomic component. In order to elucidate the protective mechanism of BA on intestinal barrier dysfunction systematically and comprehensively, the microbiota will be the focus in the future.

## 4. Materials and Methods

### 4.1. Chemicals

T-2 toxin was purchased from Pribolab Pte. Ltd. (Singapore). BA was purchased from Sigma (St. Louis, MO, USA). VE, used as positive control [56,57], was bought from Sigma-Aldrich (St Louis, MO, USA). MDA, GSH, SOD, GSH-PX and CAT assay kits were acquired from Nanjing Jiancheng Biotech (Nanjing, Jiangsu, China). The H&E staining solution was purchased from Servicebio (Wuhan, Hubei, China). IgG, SIgA, IgM, C3, C4 and DAO kits were supplied from Elabscience Biotechnology Co., Ltd. (Wuhan, Hubei, China). SYBR Green I fluorescent dyes and the Primescript RT reagent kit were from Takara (Shiga, Japan) and trizol was supplied by Life Technologies (Carlsbad, CA, USA). The IKB-α antibody, NF-κB p65 antibody and β-actin antibody were bought from Cell Signaling Technology, Inc. (Danvers, MA, USA), and all other reagents were analytical grade.

### 4.2. Animals and Experimental Designs

Sixty specific pathogen-free male Kunming mice weighing 20 ± 2 g were provided by Hunan Silaikejingda Laboratory Animal Co., Ltd. (Changsha, Hunan, China). The mice were acclimated in a temperature (22–25 °C) and humidity-controlled (50–70%) room in an adequately ventilated laboratory, with light and dark cycles of 12 h and free access to water and food. The administration time and the doses of T-2 toxin and BA were optimized on the basis of previous studies [57,58]. Mice were randomly assigned to six groups, namely, the control group, T-2 toxin only group, low-, medium- and high-dose BA (0.25, 0.5 or 1 mg/kg bw) and T-2 toxin cotreatment groups and the VE and T-2 toxin cotreatment group. VE and BA were suspended in 1% soluble starch and given orally daily for 2 weeks. At the same time, the control group and T-2 toxin only group were administered the same amount of 1% soluble starch. Then, 4 mg/kg T-2 toxin, dissolved in a mixed solution of alcohol and PBS (alcohol/PBS = 1:12.5), was injected intraperitoneally to induce oxidative damage, while the control group was injected with an equal volume of the mixed solution of alcohol and PBS. Serum samples were collected according to a previous method [58]. Mice were humanely sacrificed and the jejunum was quickly excised and stored at −80 °C until analysis.

### 4.3. Detection of MDA, CAT, GSH and GSH-PX Levels in the Jejunum

Tissues were homogenized in cold physiological saline to yield a 10% *w*/*v* jejunum homogenate. The homogenized tissues were centrifuged at 3000 rpm for 15 min at 4 °C. The supernatant produced after processing jejunum samples was separated and used for further biochemical assessments. The antioxidase activities of GSH-PX and CAT, reduced GSH level and the content of MDA as a marker of lipid peroxidation were estimated using the assay kits and strictly following the instructions.

### 4.4. Histological and Ultrastructural Observations

Jejunum tissues were dehydrated and embedded in paraffin after fixing with 10% neutral-buffered paraformaldehyde. All sections were cut into a thickness of approximately 5 μm, stained with hematoxylin and eosin (Servicebio Co., Ltd., Wuhan, Hubei, China) and observed under an optical microscope (Olympus, Tokyo, Japan) using an image capturing software (Nikon Eclipse Ci, Tokyo, Japan).

At 4 °C, the fresh jejunum samples were quickly put into 2.5% glutaraldehyde for fixation for 4 h. The samples were taken out and washed 3 times with 0.1 M PBS (pH 7.4) and then fixed with 1% osmium tetroxide for 2 h at room temperature (20 °C). After dehydration in graded ethanol solutions (50%, 70%, 80%, 90%, 95% and 100%) and acetone, the samples were embedded in the epoxy resin Epon 812 for polymerization. Then, the samples were cut into ultrathin slices (70 nm) and the slices were stained with uranyl acetate and lead citrate for 15 min. Ultrastructural changes of the jejunum were observed and photographed using a transmission electron microscope (H-7500, Hitachi, Tokyo, Japan) [17].

### 4.5. Enzyme-Linked Immunosorbent Assay (ELISA)

The secretions of SIgA, C3 and C4 in the intestine, and the levels of IgG, IgM and DAO in serum were estimated according to instructions of ELISA kits.

### 4.6. qPCR

The total RNA was extracted using the trizol reagent after homogenizing the tissue samples. Using the Primescript RT reagent kit to reverse the total RNA into cDNAs, the template cDNAs were obtained for qPCR detection. The inflammatory cytokins and TJs mRNA expressions were detected using a PremixTaq^TM^ kit and a quantitative PCR instrument (ABI Step One). The target gene expression was normalized to β-actin as an internal reference. The 2^⁻△△Ct^ method was used to calculate the mRNA expression [17]. Primers were designed according to the requirements of quantitative fluorescence PCR and synthesized by Shanghai Shenggong Biotechnology Co., Ltd. Primer-related information is shown in Table 1.

### 4.7. Western Blot Analysis

The process of Western blot was conducted according to a previous method [58]. The total proteins of jejunum samples were received using a standard protocol by lysing tissues with RIPA buffer containing phenylmethanesulfonyl fluoride (PMSF). The protein concentrations in the samples were quantified using the BCA Protein Assay Kit (Nanjing, Jiangsu, China). The jejunum proteins were separated using sodium dodecyl sulfate polyacrylamide gel electrophoresis (SDS-PAGE) and then transferred onto a PVDF membrane. The membranes were blocked with 5% defatted milk powder for 1 h and sequentially incubated with the following primary antibodies at 4 °C overnight: β-actin (diluted 1:1000), NF-κB (diluted 1:1000), IKB-α (diluted 1:1000), p-NF-κB (diluted 1:1000) and p-IKB-α (diluted 1:1000). Membranes were then incubated with a secondary antibody (diluted 1:3000) for 2 h. Protein bands were visualized using enhanced chemiluminescence (ECL) reagents (Jiangsu Keygen Biotech Corp., Ltd., Nanjing, China) and the band intensities were analyzed using Image Lab™ Software (Bio-Rad, Hercules, CA, USA).

### 4.8. Statistical Analysis

All analyses are presented as the means ± SD and estimated using Student’s t-test and one-way ANOVA, followed by Tukey’s multiple comparisons test for multiple comparisons. SPSS software (SPSS Inc., Chicago, IL, USA, 2008) for Windows version 17.0 was selected to perform all statistical analyses. *p* < 0.05 was considered a statistically significant difference.

## Figures and Tables

**Table 1:** RT-PCR primer list.

Gene	Primer Sequence (5′–3′)	
	Forward Primer	Reverse Primer
β-actin	5′-CATCCGTAAAGACCTCTATGCCAAC-3′	5′-ATGGAGCCACCGATCCACA-3′
ZO-1	5′-TACCTCTTGAGCCTTGAACTT-3′	5′-CGTGCTGATGTGCCATAATA-3′
Occludin	5′-GTGTGGTTGATCCCCAGGAG-3′	5′-TCGCTTGCCATTCACTTTGC-3′
IL-1β	5′-TGCCACCTTTTGACAGTGATG-3′	5′-TGATGTGCTGCTGCGAGATT-3′
IL-6	5′-TGATGGATGCTACCAAACTGGA-3′	5′-TGTGACTCCAGCTTATCTCTTGG-3′
IL-10	5′-GGTTGCCAAGCCTTATCGGA-3′	5′-TCAGCTTCTCACCCAGGGAA-3′
TNF-α	5′-AGCCGATGGGTTGTACCTTG-3′	5′-AGTACTTGGGCAGATTGACCTC-3′

## Abbreviations

BA	betulinic acid
IgG	immunoglobulin G
IgM	immunoglobulin M
SIgA	secretory immunoglobulin A
C	complement
IL	interleukin
TJs	tight junction proteins
NF-κB	nuclear factor-kappa B
